# Stem cell properties *of peripheral blood endothelial progenitors are stimulated by soluble CD146 via miR-21: potential use in autologous cell therapy*

**DOI:** 10.1038/s41598-018-27715-4

**Published:** 2018-06-20

**Authors:** Amel Essaadi, Marie Nollet, Anaïs Moyon, Jimmy Stalin, Stéphanie Simoncini, Laure Balasse, Alexandrine Bertaud, Richard Bachelier, Aurélie S. Leroyer, Gabrielle Sarlon, Benjamin Guillet, Françoise Dignat-George, Nathalie Bardin, Marcel Blot-Chabaud

**Affiliations:** 1 Aix Marseille Univ, INSERM 1263, INRA 1260, C2VN Marseille, France; 2 CERIMED (European Center of Research in Medical Imaging), Aix-Marseille University, Marseille, France; 3 Service of Vascular Surgery, La Timone Hospital, Marseille, France

## Abstract

Cell-based therapies constitute a real hope for the treatment of ischaemic diseases. One of the sources of endothelial progenitors for autologous cell therapy is Endothelial Colony Forming Cells (ECFC) that can be isolated from peripheral blood. However, their use is limited by their low number in the bloodstream and the loss of their stem cell phenotype associated with the acquisition of a senescent phenotype in culture. We hypothesized that adding soluble CD146, a novel endothelial growth factor with angiogenic properties, during the isolation and growth procedures could improve their number and therapeutic potential. Soluble CD146 increased the number of isolated peripheral blood ECFC colonies and lowered their onset time. It prevented cellular senescence, induced a partial mesenchymal phenotype and maintained a stem cell phenotype by stimulating the expression of embryonic transcription factors. These different effects were mediated through the induction of mature miR-21. When injected in an animal model of hindlimb ischaemia, sCD146-primed ECFC isolated from 40 ml of blood from patients with peripheral arterial disease were able to generate new blood vessels and restore blood flow. Treatment with sCD146 could thus constitute a promising strategy to improve the use of autologous cells for the treatment of ischaemic diseases.

## Introduction

Ischaemic diseases are a major cause of mortality in the world. The recent discovery that vascular progenitor cells can regenerate functional blood vessels has raised great hope^1^ and cell-based therapies have emerged as a promising approach for their treatment. Along this line, several clinical trials based on autologous bone marrow-derived cells or mesenchymal stem cells injection have been performed^2^. However, these cell therapy products are heterogeneous in composition and only a few cells involved in vascular regeneration attain the ischaemic area, leading to disappointing results. Another strategy consists of the generation of a homogeneous cell therapy product composed of endothelial cells, the Endothelial Colony Forming Cells (ECFC). These cells can be isolated from peripheral blood and amplified in culture before injection into patients^3^. However, their use, especially in patients with vascular pathologies, is limited by their low number in the bloodstream, the technical difficulties of isolation and growth *in vitro* and the loss of their stem cell phenotype coupled to a senescent phenotype in culture.

CD146 is a cell adhesion molecule belonging to the immunoglobulin superfamily that was recently shown to be present on endothelial cells and to be involved in angiogenesis^4^. The shedding of CD146 leads to the secretion of a soluble form (sCD146) that constitutes a new growth factor stimulating angiogenesis *in vitro* and *in vivo*^5^. In a recent study, we have described that priming of ECFC obtained from cord blood with sCD146 improved their survival capacity *in situ* and their angiogenic properties. Of interest, we showed that these effects involved both the short isoform of CD146 and the VEGFR1/VEGFR2 pathways^6^. Soluble CD146 binding on its receptor angiomotin activated the proteolytic processing of the short isoform of CD146, leading to the generation of an intracellular CD146 fragment that was targeted toward the nucleus and induced the transcription of genes, including transcription factors^6^. This proteolytic processing has already been described for Notch^7^. Of interest, the Notch signalling pathway is involved in the regulation of many cellular properties, including cell death, senescence and stem cell properties. This has been especially demonstrated in cancer where Notch controls the generation of cancer stem cells^8^. In these cells, it acts through the modulation of a large miRNA network^9^.

In view of the angiogenic properties of sCD146 and of the similarities in the proteolytic processing of Notch and the short CD146 isoform, we hypothesized that sCD146 could constitute a factor able to stimulate the stem cell phenotype and decrease the senescent phenotype of peripheral blood ECFC. This could therefore be of potential interest for their amplification in culture before autologous re-injection to patients. We thus addressed the effects of sCD146 on 1/peripheral blood ECFC sorting efficacy; 2/peripheral blood ECFC stem cell properties and senescence; 3/peripheral blood ECFC miRNA expression and 4/peripheral blood ECFC regenerative properties *in vivo*. In view of the promising results, the molecule was tested on the sorting and regenerative capacity of ECFC isolated from peripheral blood of patients with peripheral arterial disease.

## Results

### Isolating ECFC from peripheral blood in the presence of soluble CD146 enhances the number of colonies, lowers their onset time and boosts their regenerative properties *in vivo*

**Figure 1:**
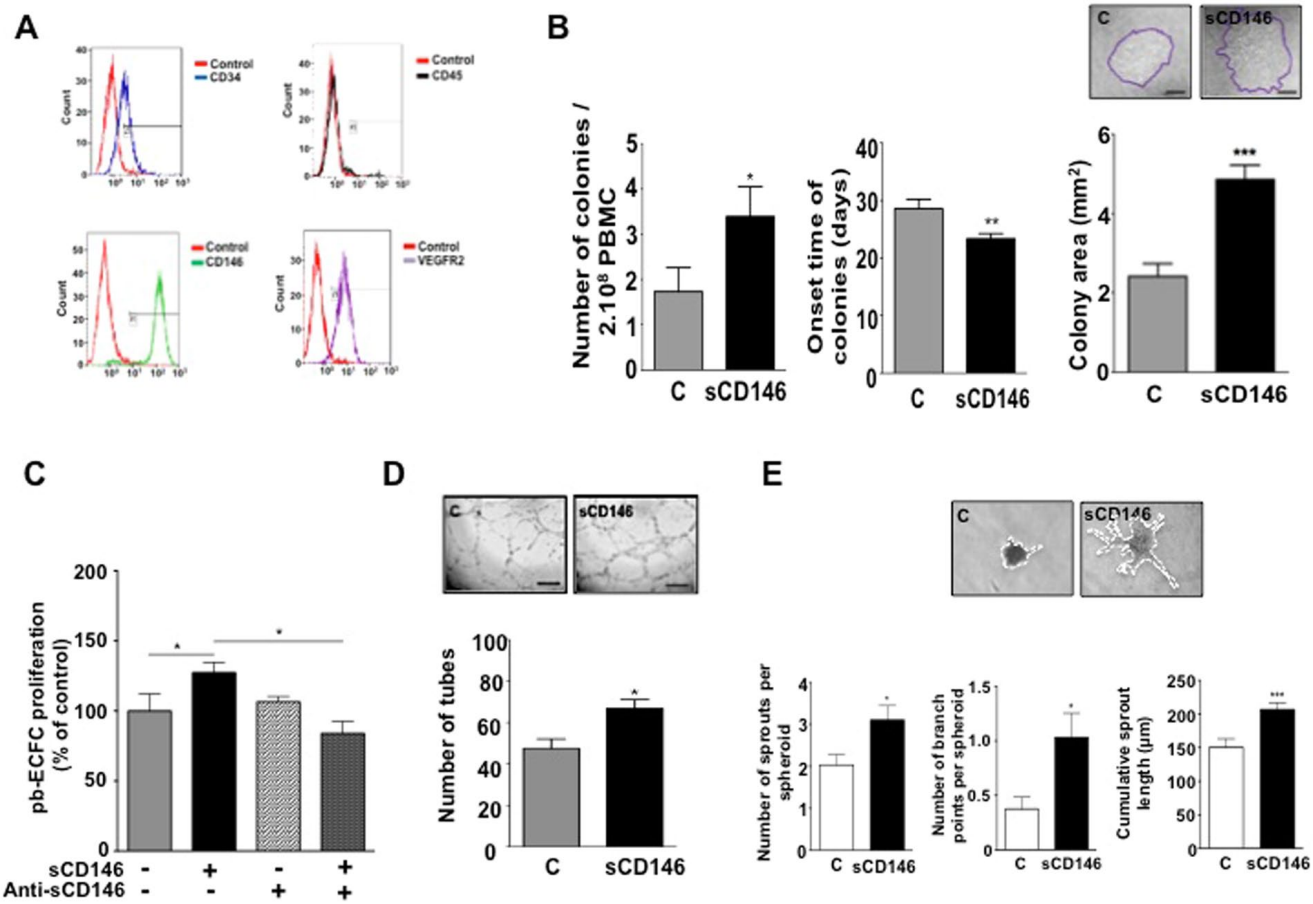
Soluble CD146 enhances the number and proliferation of ECFC colonies isolated from peripheral blood of patients and lowers their onset time. (**A**) ECFC derived from peripheral blood of patients were characterized by FACS. ECFC display an endothelial phenotype characterized by the expression of CD34, no expression of CD45, and expression of both CD146 and VEGFR2. Plots are representative of 3 independent experiments. (**B**) The number of ECFC colonies and their time of onset were determined when the isolation procedure was performed in the absence or presence of 50 ng/ml sCD146. The surface area of each colony was also determined at day 25. Results are mean values +/− SEM of 16 independent experiments. (**C**) Proliferation of ECFC was determined in the absence or presence of sCD146. An anti-sCD146 antibody (M2J-1 mAb) was used to test the specificity of the effect. The results are mean values +/− SEM of 5 independent experiments. (**D**) The number of tubes was determined in a Matrigel assay when ECFC were incubated in the absence or presence of 50 ng/ml sCD146. Pictures are representative of 10 independent experiments. (**E**) The number of sprouts, number of branch points and cumulative sprout length were determined in spheroid assays when ECFC were incubated or not in the presence of 50 ng/ml sCD146. Pictures are representative of 10 independent experiments. *P < 0.05, **P < 0.01, ***P < 0.001, experimental vs. Control.

To study the influence of soluble CD146 (sCD146) on the isolation of ECFC from peripheral blood (pb-ECFC), we performed experiments in which ECFC were isolated in the presence or in absence of 50 ng/mL recombinant sCD146 from the beginning of the experiment (cells laid out in culture) and thereafter changed every 2 days with the medium.

pb-ECFC were first characterized by flow cytometry analysis to verify that the ECFC obtained were not contaminated by other cell types. The results show that cells expressed CD146, VEGFR2, and CD34 at a high level and did not express CD45 (Fig. 1A). The effect of sCD146 treatment was also studied in terms of cell markers expression. The results (Supplementary Fig. 1A and B) show that treatment with the molecule induced an increase in mRNA and protein expression of CD146 and VEGFR2, but also of the receptor of sCD146, angiomotin.

Then, the number of colonies, their onset time and the surface of expansion of colonies observed at day 25 were analysed in the pb-ECFC compared to pb-ECFC treated with sCD146 (pb-ECFC-sCD146). The number of colonies was significantly increased by approximately twofold and their onset time was significantly reduced of approximately 5 days in the pb-ECFC-sCD146 compared to pb-ECFC. In addition, the mean surface covered by colonies, and thus the total number of growing cells was significantly increased in pb-ECFC-sCD146 compared to pb-ECFC (Fig. 1B).

To confirm the high proliferative capacity of pb-ECFC grown in the presence of sCD146 and to analyse the ability of the cells to form capillary-like structures, we performed proliferation experiments as well as matrigel and spheroid assays. As shown by the BrdU incorporation assay, pb-ECFC-sCD146 exhibited a significantly higher proliferation rate than pb-ECFC. When sCD146 was blocked with an anti-sCD146 specific antibody (M2J-1 mAb) in pb-ECFC-sCD146, proliferation was equivalent to that of pb-ECFC (Fig. 1C). Of interest, the proliferative capacity of pb-ECFC-sCD146 was increased compared to pb-ECFC in the long term. Indeed, even if the proliferative capacities of both pb-ECFC and pb-ECFC-sCD146 decreased with the number of passages, pb-ECFC-sCD146 remained significantly more proliferative than pb-ECFC up to passage 5 (Supplementary Fig. 2). In Matrigel assays, pretreatment of pb-ECFC with sCD146 significantly increased the formation of endothelial networks, as shown by an increased number of closed tubes (Fig. 1D). In addition, in a three-dimensional spheroid assay, sprout formation, length and branching were also increased by sCD146 treatment (Fig. 1E).

**Figure 2:**
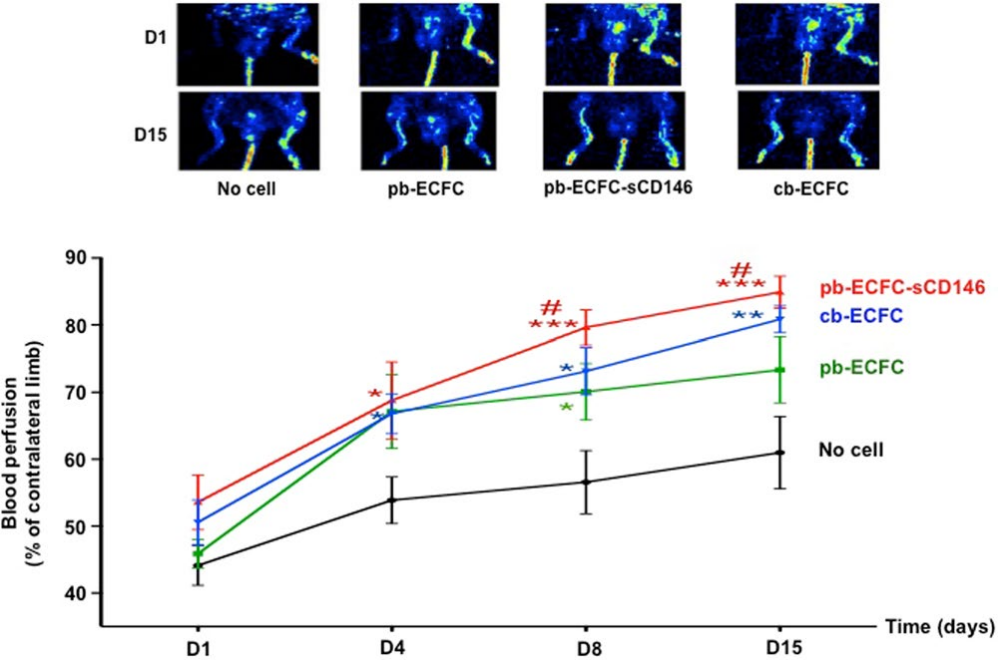
Soluble CD146 enhances regenerative properties of ECFC from peripheral blood in an animal model of hindlimb ischaemia. Blood perfusion rate was determined by laser-doppler in hindlimb of nude mice with ischaemia. Animals were injected at day 1 with PBS, ECFC from peripheral blood, or ECFC from peripheral blood obtained in the presence of 50 ng/ml sCD146 or ECFC from cord blood. Blood perfusion was determined in the ischaemic hindlimb and expressed as a % of the blood perfusion measured in the contra-lateral limb. Representative pictures of laser-doppler are shown and mean values +/− SE of 8 animals are given. *^,^**^,^***P < 0.05, P < 0.01, P < 0.001, experimental *vs* no cell at each time. ^#^P < 0.05, pbECFC-sCD146vspbECFC.

In view of the angiogenic properties of pb-ECFC observed *in vitro*, we performed *in vivo* experiments in a model of Nude mice with hindlimb ischaemia. Animals were injected with 250,000 ECFC obtained from cord blood (cb-ECFC) or peripheral blood (grown with or without sCD146) and compared to mice without cell injection (control mice). The blood perfusion rate was then determined at days 1, 4, 8 and 15 after surgery by laser doppler. The results show that injection of ECFC from both cord and peripheral blood significantly increased the blood perfusion rate from day 8 compared to control mice. When pb-ECFC were grown in the presence of sCD146, the blood perfusion rate was similar to that observed in cb-ECFC (Fig. 2).

### Soluble CD146 stimulates stem cell phenotype in ECFC isolated from peripheral blood

**Figure 3:**
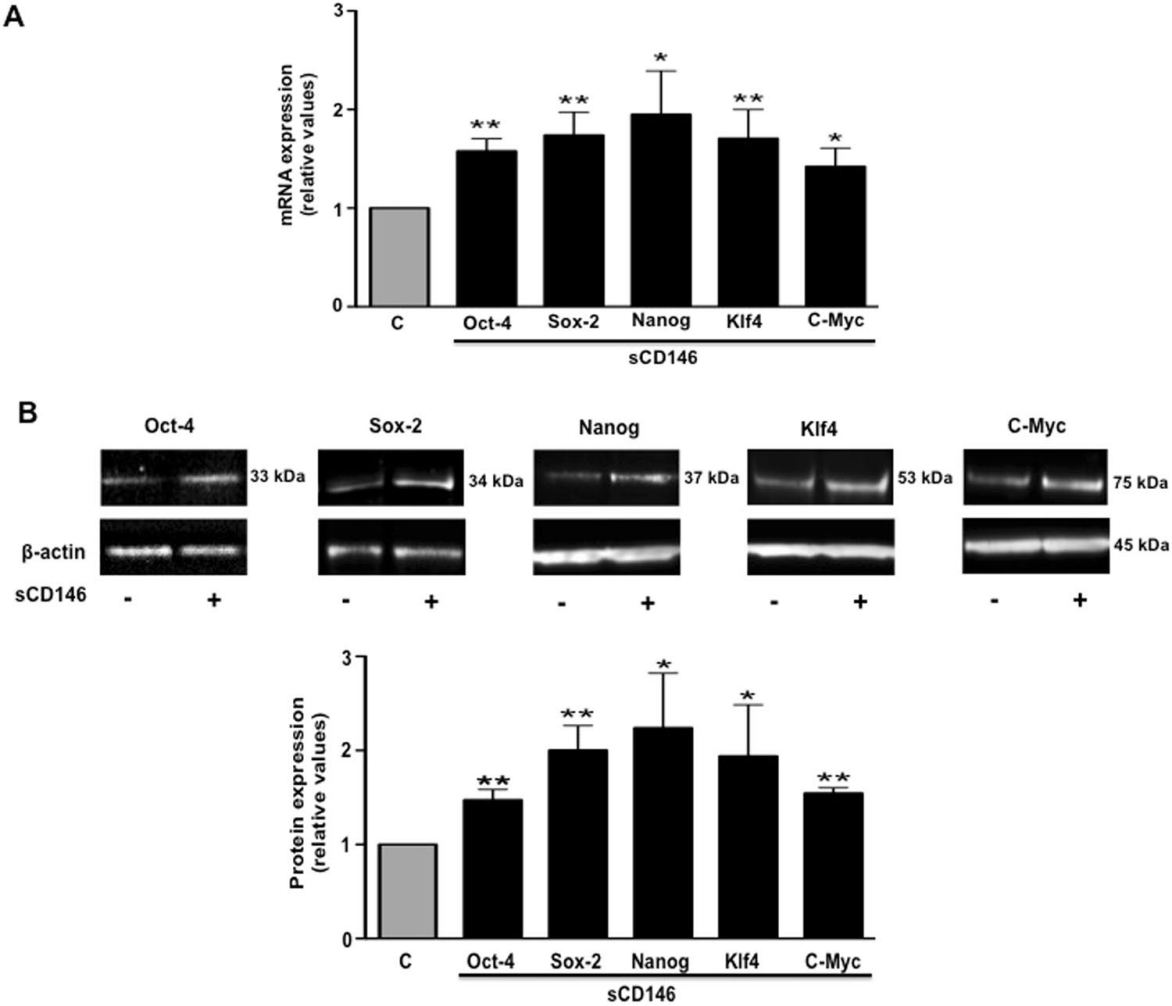
Soluble CD146 increases embryonic transcription factors in ECFC isolated from peripheral blood of patients. (**A**) Effect of 48 hours of treatment with 50 ng/ml sCD146 on the mRNA expression of Oct-4, Sox-2, Nanog, KLF-4 and c-myc. (**B**) Effect of 48 hours of treatment with 50 ng/ml sCD146 on the protein expression of Oct-4, Sox-2, Nanog, KLF-4 and c-myc. Pictures are representative and results are the mean values +/−  SEM of 6 independent experiments. *P < 0.05, **P < 0.01, experimental vs. Control. To improve the clarity and conciseness of the figure, cropped gels corresponding to the band of interest are shown. β-actin was visualized and analysed on the same gel. Quantitative comparisons between samples were performed on samples migrated on the same gels.

As it is known that the expression of embryonic factors modulates the differentiation of eukaryote cells, we investigated the impact of sCD146 on 5 embryonic transcription factors. The results show a significant increase in mRNA expression of Oct-4, Sox-2, Nanog, Klf-4 and c-Myc markers in pb-ECFC treated for 48 hours with 50 ng/ml sCD146, as compared to pb-ECFC from the same clone (Fig. 3A). These results were confirmed at the protein level by western blotting (Fig. 3B).

**Figure 4:**
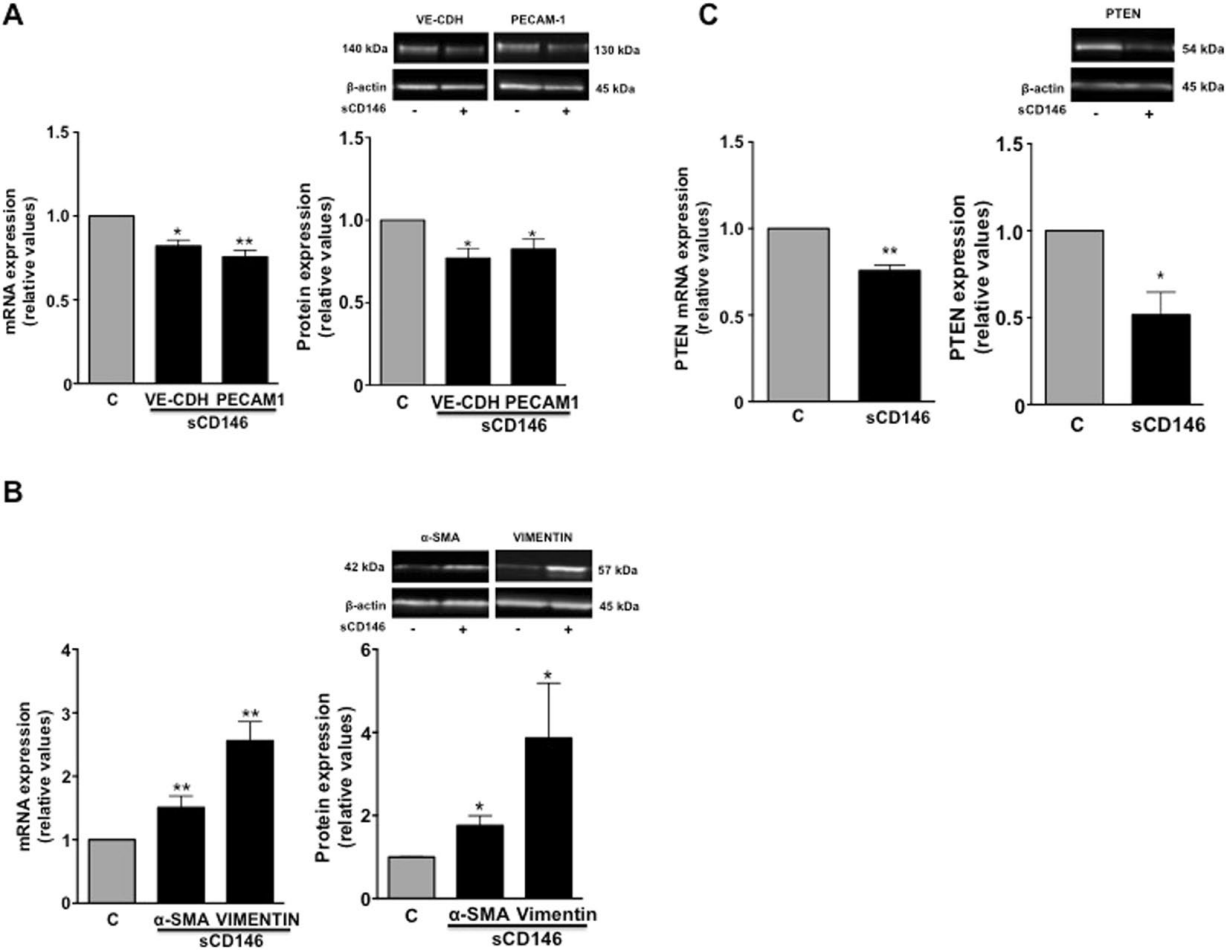
Soluble CD146 induces a partial endothelial-mesenchymal transition in ECFC isolated from peripheral blood of patients. (**A**) Effect of 48 hours of treatment with 50 ng/ml sCD146 on the mRNA and protein expression of VE-cadherin (VE-CDH)and PECAM-1. (**B**) Effect of 48 hours of treatment with 50 ng/ml sCD146 on the mRNA and protein expressions of α-SMA and vimentin. (**C**) Effect of 48 hours of treatment with 50 ng/ml sCD146 on the mRNA and protein expressions of PTEN. Pictures are representative, and results are the mean values +/−  SEM of 4–5 independent experiments. *P < 0.05, **P < 0.01, experimental vs. Control. To improve the clarity and conciseness of the figure, cropped gels corresponding to the band of interest are shown. β-actin was visualized and analysed on the same gel. Quantitative comparisons between samples were performed on samples migrated on the same gels.

It was recently reported that CD146 is involved in the epithelial-to-mesenchymal transition (EMT). However, its influence on the endothelial-to-mesenchymal transition (EndoMT) has never been studied. We thus tested whether sCD146 could influence EndoMT in pb-ECFC. To this end, we evaluated the mRNA and protein expression of mesenchymal *versus* endothelial markers in pb-ECFC treated for 48 hours with sCD146 50 ng/ml compared to pb-ECFC from the same clone. In addition, we analysed the expression of PTEN since it is a negative regulator of AKT and constitutes the main pathway involved in the EndoMT process. The results show that sCD146 decreased VE-cadherin and PECAM-1 expression and increased α-SMA and vimentin, both at the mRNA and protein levels (Fig. 4A,B). Concerning PTEN, its expression was down-regulated by sCD146, both at the mRNA and protein levels (Fig. 4C).

### Soluble CD146 prevents cellular senescence in ECFC isolated from peripheral blood

**Figure 5:**
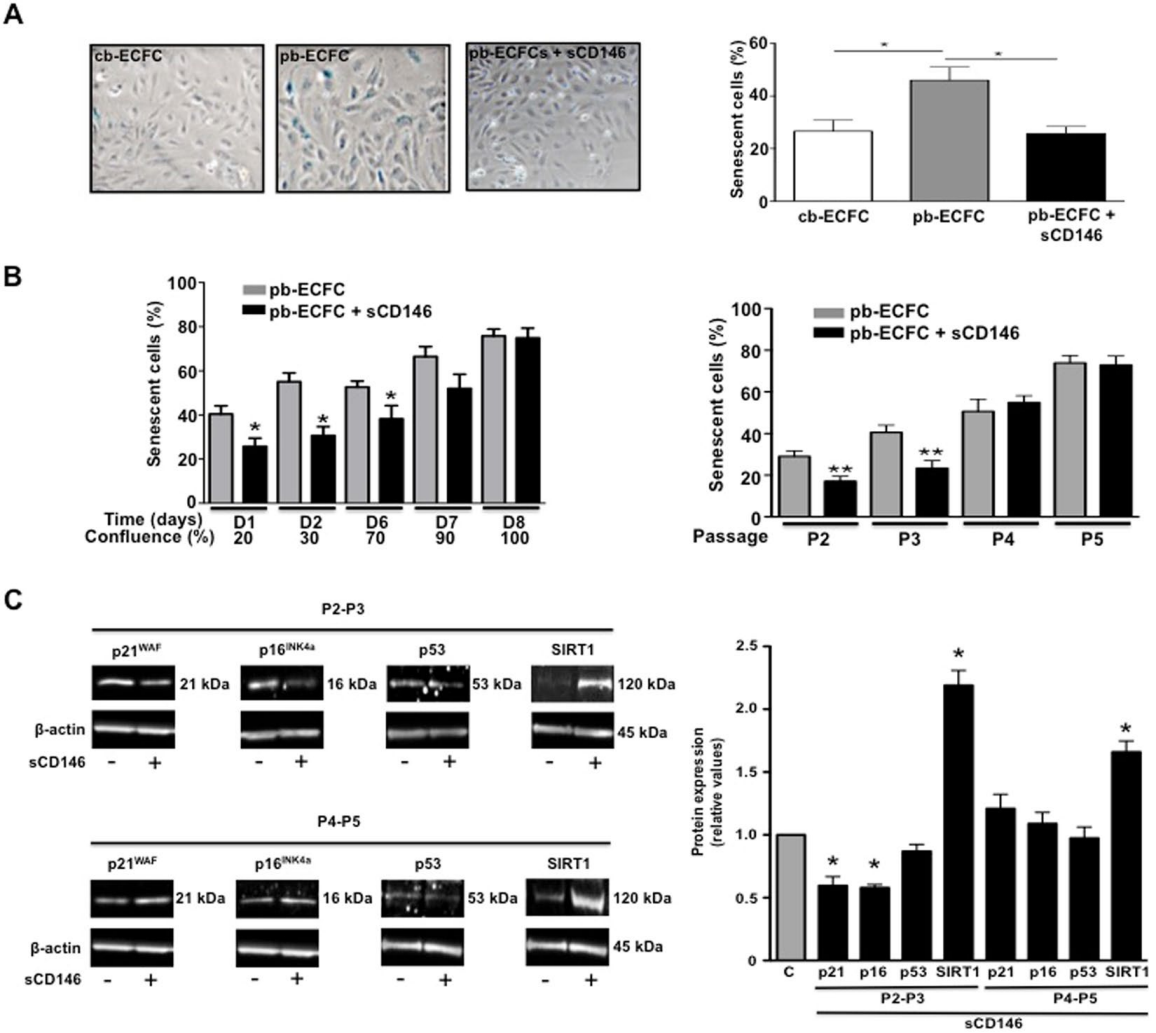
Soluble CD146 decreases senescence in ECFC isolated from peripheral blood of patients. (**A**) Effect of 48 hours of treatment with 50 ng/ml sCD146 on the senescence of pb-ECFC and comparison with cb-ECFC. Representative pictures are shown. The results are the mean values +/−  SEM of 4 different experiments. (**B**) The ratio of senescent cells to the total number of cells is given in pb-ECFC and pb-ECFC treated for 48 h with 50 ng/ml sCD146 as a function of the % of confluence of the cells (left panel) and of the number of passages of the cells (right panel). The results are the mean values +/−  SEM of 9–11 independent experiments. (**C**) Effect of 48 h of treatment with 50 ng/ml sCD146 on the protein expression of p21^WAF^, p16^INK4a^, p53 and SIRT1 in pb-ECFC at passages 2–3 and passages 4–5. The same actin was used for p53 and sirt1 because the western-blot membrane was cut in three parts for revealing with the three antibodies. Representative pictures are shown, and mean values +/− SE of 4 experiments are given. *P < 0.05, **P < 0.01, experimental vs. Control. To improve the clarity and conciseness of the figure, cropped gels corresponding to the band of interest are shown. β-actin was visualized and analysed on the same gel. Quantitative comparisons between samples were performed on samples migrated on the same gels.

We compared the senescence of cb-ECFC and pb-ECFC under different conditions. Using SA-β-galactosidase staining, we observed that sCD146 decreased the senescence of pb-ECFC (at approximately 70% confluence) and restored it at a level similar to that of cb-ECFC (Fig. 5A). Whatever the cell type, cellular senescence increased with the confluence of cells and with the number of passages in culture. The results also showed that over 70% confluence (90–100% confluence) and over passage 3, no difference was observed in senescence between pb-ECFC and pb-ECFC treated for 48 h with 50 ng/ml sCD146 (Fig. 5B). The major pathways controlling senescence converge at cyclin-dependent kinase inhibitor p16^INK4a^ and p21^WAF^^10,11^. Therefore, we assessed the expression of these cell cycle markers by immunoblotting. The protein expression of p21^WAF^ and p16^INK4a^ was significantly decreased in pb-ECFC treated with sCD146 compared to pb-ECFC when cells were used at early passages (2–3) (Fig. 5C). In contrast, p53 protein expression of pb-ECFC was not modulated by the pretreatment with sCD146 whatever the passage. We also studied whether, in pb-ECFC, sCD146 could modulate SIRT1, a protein considered as a regulator of longevity or ageing in different organisms. We observed a significant increase in SIRT1 expression in pb-ECFC treated with sCD146 compared to non-treated pb-ECFC in both P2-P3 and P4-P5 cells (Fig. 5C).

### Soluble CD146 modulates miRNA expression in ECFC isolated from peripheral blood: coordinating role of mature miR-21

Since miRNA have been frequently reported to control differentiation and angiogenesis^12,13^, we analysed the effect of 48 hours of treatment with 50 ng/ml sCD146 on miRNA expression using a Cell-Associated miRNA Plate Array. The results show that sCD146 up-regulated different miRNAs notably mature miR-21 which was highly increased (Supplementary Fig. 3A). Several miRNAs were also down-regulated (Supplementary Fig. 3B).

**Figure 6:**
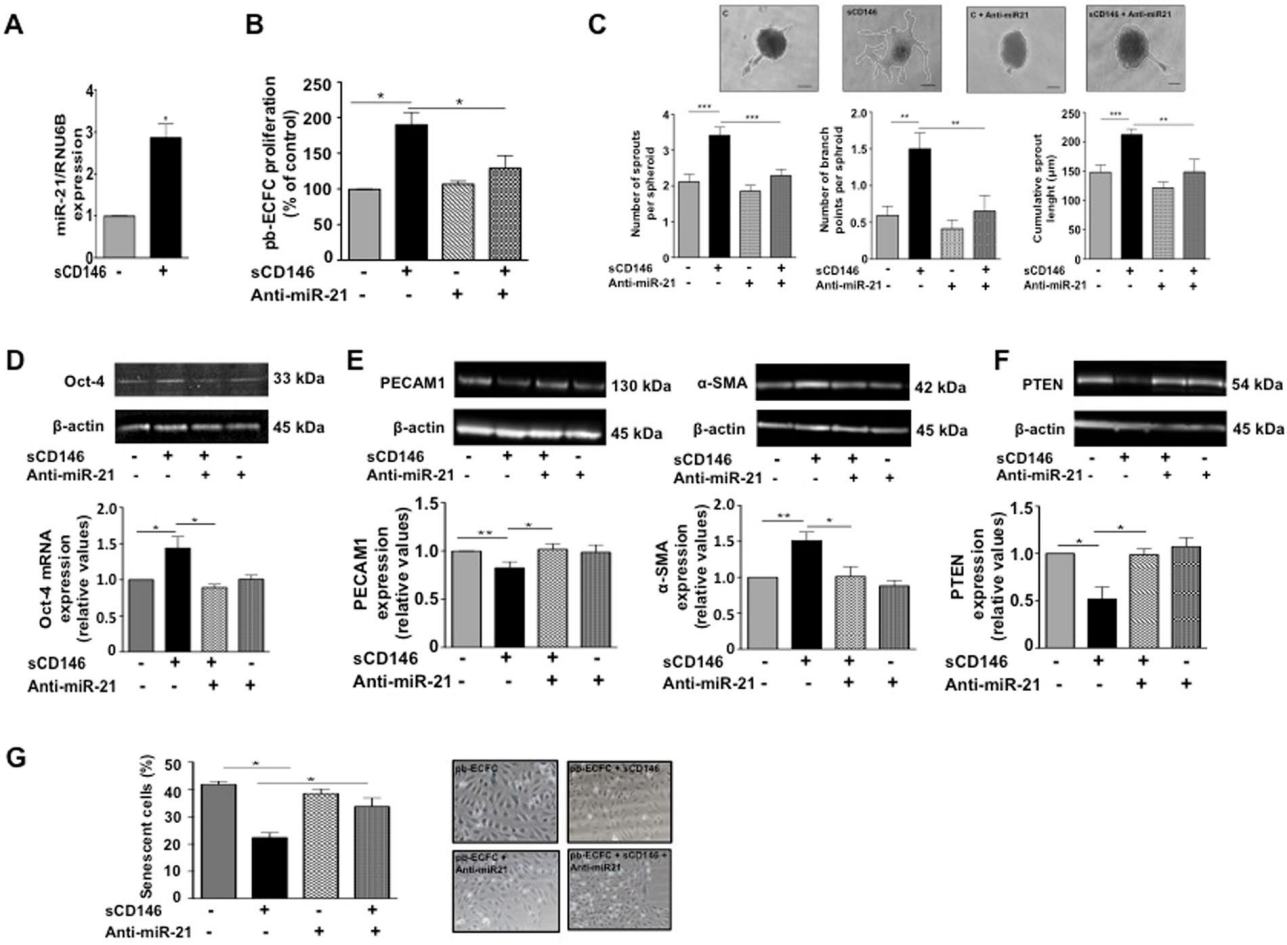
Mature miR-21 coordinates soluble CD146 effects. (**A**) Effect of 48 hours of treatment with 50 ng/ml of soluble CD146 on mature miR-21 expression in pb-ECFC. Mean values +/− SE of 4 experiments are given. (**B**) Effect of anti-miR-21 on the sCD146-induced increase in pb-ECFC proliferation (48 hours of treatment with 50 ng/ml sCD146). Mean values +/− SE of 4 experiments are given. (**C**) Effect of anti-miR-21 on the sCD146-induced increase in the number of sprouts, branch points and cumulative sprout length in spheroid assays performed with pb-ECFC (48 hours of treatment with 50 ng/ml sCD146). For each experiment, sprouts from 20 spheroids were counted. Mean values +/− SE of 6 independent experiments are given. (**D**) Effect of anti-miR-21 on the sCD146-induced increase in Oct-4 (48 hours of treatment with 50 ng/ml sCD146). Representative pictures are shown and mean values +/− SE of 4 independent experiments are given. (**E**) Effect of anti-miR-21 on the sCD146-induced modulation of PECAM-1 and α-SMA in pb-ECFC (48 hours of treatment with 50 ng/ml sCD146). Representative pictures are shown and mean values +/− SE of 5 independent experiments are given. (**F**) Effect of anti-miR-21 on the sCD146-induced decrease in PTEN in pb-ECFC (48 hours of treatment with 50 ng/ml sCD146). Representative pictures are shown and mean values +/− SE of 4 independent experiments are given. (**G**) Effect of anti-miR-21 on the sCD146-induced decrease in the number of senescent pb-ECFC (48 hours of treatment with 50 ng/ml sCD146). Representative pictures are shown and mean values +/− SE of 4 independent experiments are given. *^,^**^,^***P < 0.05, P < 0.01, P < 0.001, experimental vs. Control.

To study the influence of mature miR-21 in pb-ECFC, we transfected pb-ECFC under different conditions in the presence or absence of sCD146 and/or anti-miR-21(inhibitor of the mature miR-21 molecule). In the first step, we studied mature miR-21 expression in pb-ECFC and pb-ECFC treated with sCD146. The results confirmed that sCD146 increased the expression of mature miR-21 in pb-ECFC (Fig. 6A). We tested the role of mature miR-21 in the angiogenic properties of pb-ECFC treated with sCD146. The results show that transfection of pb-ECFC with anti-miR-21 blocked the sCD146-induced increase in cell proliferation (Fig. 6B). Likewise, it blocked the sCD146-induced increase in length and branching of sprouts in the spheroid assay (Fig. 6C).

We then investigated the role of mature miR-21 on the expression of embryonic factors and the EndMT transition. The results show that when pb-ECFC treated with sCD146 were transfected with anti-miR-21, the sCD146-induced increase in Oct-4 expression was inhibited (Fig. 6D). Likewise, when pb-ECFC were transfected with anti-miR-21, the sCD146-induced decrease in PECAM-1 and increase in α-SMA were blocked (Fig. 6E). The PTEN signalling pathway was also modulated by sCD146 in pb-ECFC via mature miR-21. Indeed, when pb-ECFC treated with sCD146 were transfected with anti-miR-21, the sCD146-induced decrease in PTEN was blocked (Fig. 6F). Finally, we studied the role of mature miR-21 in the control of senescence of pb-ECFC by sCD146. In pb-ECFC treated with sCD146 transfected with anti-miR-21, senescence was significantly increased compared to pb-ECFC only treated with sCD146, and the senescence rate was equivalent to that of pb-ECFC (Fig. 6G).

### Endothelial progenitor cells isolated from peripheral blood of patients with peripheral arterial disease (PAD) in the presence of soluble CD146 restore blood flow in ischaemic mice

In a last series of experiments, we tested the interest of sCD146 for the isolation and amplification of ECFC isolated from peripheral blood of patients with PAD. The clinical characteristics of the patients are described in the Material and Methods section and in Supplementary Table 1. The results show that it was possible to isolate ECFC from the peripheral blood of these patients, to amplify them *in vitro* and to restore blood flow in an animal model of hindlimb ischaemia after re-injection. Indeed, among 4 patients, we were able to isolate one colony from 40 ml of peripheral blood in three of the four patients. This colony appeared at an onset time between 16 and 23 days, a time similar to that obtained for control patients (see Fig. 1B). This colony could be grown *in vitro* to generate >100,000 cells after 30 days of culture with sCD146.

**Figure 7:**
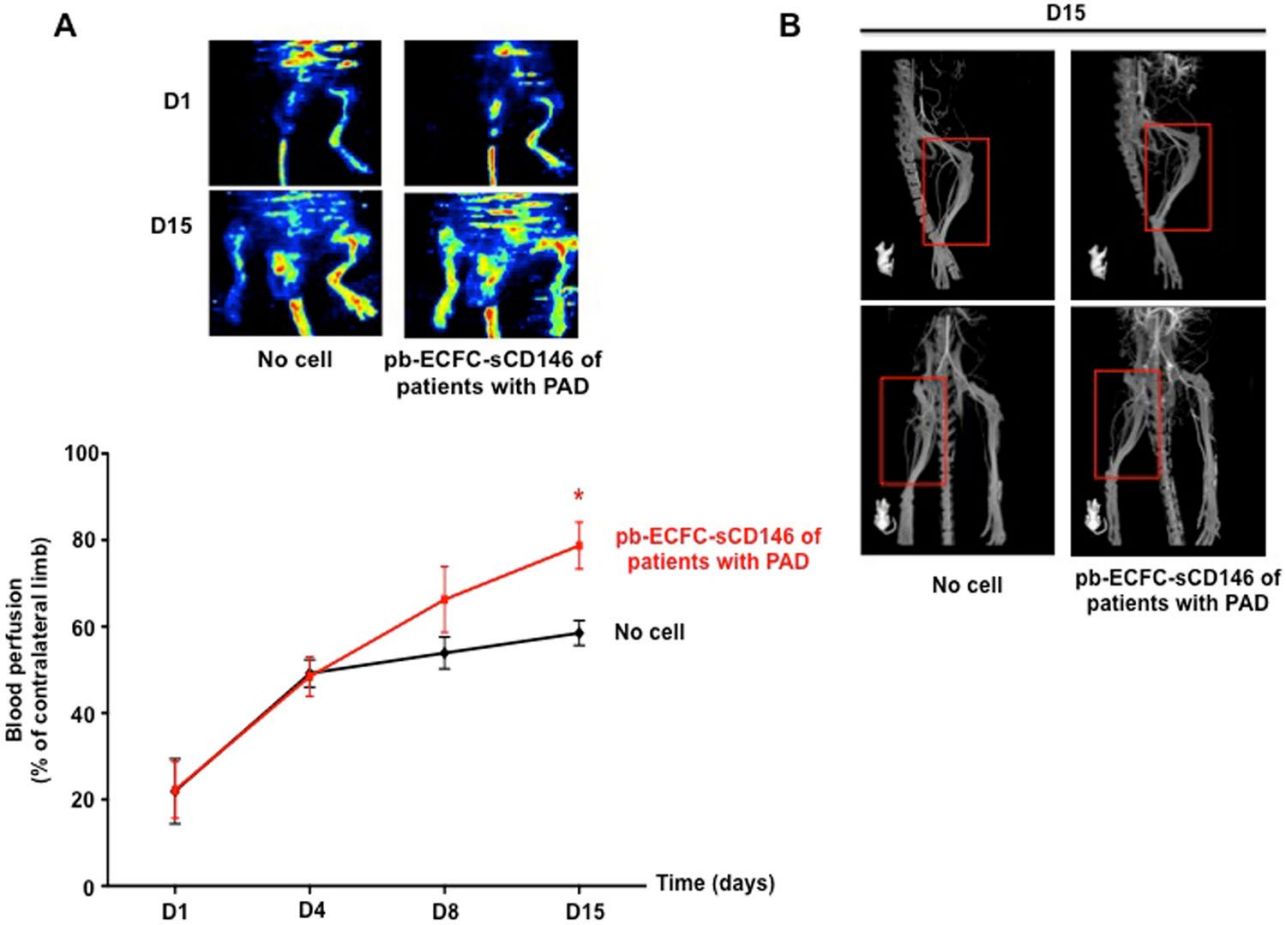
ECFC from peripheral blood of patients with PAD isolated in the presence of sCD146 restore blood flow in an animal model of hindlimb ischaemia. (**A**) Blood perfusion rate was determined by laser-doppler in hindlimb of nude mice with ischaemia. Animals were injected at day 1 with PBS or ECFC from peripheral blood of patients with PAD isolated in the presence of 50 ng/ml sCD146. Blood perfusion was determined in the ischaemic hindlimb and expressed as a % of the blood perfusion measured in the contralateral limb. Representative pictures of laser-doppler are shown and mean values +/− SE of 3 patients are given. *P < 0.05, pbECFC-sCD146 versus no cell. (**B**) Angiography experiments were performed at day 15. One representative experiment of the three experiments is shown.

Indeed, blood perfusion was significantly increased at day 15 when nude mice were treated with 100,000 sCD146-sorted ECFC compared to mice without cell injection (Fig. 7A). We also carried out angiography experiments at day 15. The images show a better collateral re-vascularisation in animal receiving cells compared to animals without cell injection, corroborating the results obtained by laser-doppler (Fig. 7B).

## Discussion

Although injection of endothelial progenitor cells (EPC) could constitute a good therapeutic approach to increase blood perfusion in ischaemic tissues, the ability to obtain EPC from peripheral blood of patients and to maintain these EPC with a non-senescent phenotype and with a low differentiation state during the expansion procedure *in vitro* remains challenging. Different growth factors have been proposed to prime EPC as a new strategy to enhance their cell therapy potential. Thus, priming of ECFC with EPO or SDF-1 has been described to significantly improve the angiogenic properties of these cells^14,15^. In a recent study, we have reported that priming ECFC obtained from cord blood with soluble CD146 (sCD146) enhanced their survival and regenerative properties both *in vitro* and *in vivo*. The observed effects were dependent upon the short isoform of CD146 (shCD146) and involved two successive shedding mechanisms. Soluble CD146 (sCD146) induced sequentially the shedding of the extracellular and intracellular parts of shCD146, leading to the generation of an intracellular domain (shCD146-IC). This shCD146-ICD was able to translocate into the nucleus to induce the transcription of genes. Among them, genes involved in cell survival and angiogenesis were identified^6^. Different proteins have been reported to act through this proteolytic processing, such as CD44 or Notch. Of interest, these two proteins are involved in the regulation of cell differentiation^16–18^. The observed similarities in the mechanisms of action of the molecules led us to investigate the influence of soluble CD146 on the stem cell properties of ECFC derived from peripheral blood and the ability to generate and expand colonies of ECFC. In this study, we show that addition of sCD146 throughout isolation and expansion led to a significantly increased probability of generating colonies with high proliferation capacity. This could involve an effect of sCD146 on the adhesion capacity of the cells. Of interest, we also show that treatment with sCD146 maintains the stem cell properties of ECFC from peripheral blood in such a way that they display regenerative properties very similar to that of cord blood-derived ECFC. Thus, their ability to restore blood perfusion in an animal model of hindlimb ischaemia is comparable. This stem cell phenotype was also attested by the sCD146 induction of embryonic genes such as oct-4, sox-2, c-myc, nanog and Kfl-4, both at the mRNA and protein levels. Of interest, four of these genes, oct-4, sox-2, c-Myc and Kfl-4, constitute the Yamanaka factors which allow somatic cell reprogramming to induce pluripotent stem cells (iPS)^19^. The gene expression and chromatin state of iPS were found to be very similar to their embryonic stem (ES) cell counterparts. The iPS constitute a promising way to regenerate tissues due to the ease of obtaining primary cells that are genetically reprogrammed and easy to expand. Although the levels of induction of these factors by sCD146 in ECFC are much lower than that observed in iPS, we can assume that they participate in the induction of a stem cell phenotype. This is also demonstrated by the partial acquisition of a mesenchymal phenotype by ECFC. Thus, our results show that treatment with sCD146 induced a reduction in the endothelial markers Pecam-1 and VE-cadherin and an increase in the mesenchymal markers α-SMA and vimentin. Other proteins, such as angiomotin or VEGFR2, which have been shown to prevent cell differentiation in different cellular models, or to be markers of stem cells, are increased^20–23^. In our experiments, we also showed that PTEN was down-regulated by sCD146. PTEN is a negative regulator of PI3K/AKT pathways which are involved in the epithelial-mesenchymal transition (EMT). In addition, PTEN has been shown to be modulated by miRNA in many studies. Thus, the overexpression of miRNA-92a enhanced EMT in non-small cell lung cancer by directly targeting PTEN^24^. Likewise, miR-216a promotes the metastasis and EMT of ovarian cancer by suppressing the PTEN/AKT pathway^25^. The miRNA-221/222 also promotes cancer stem-like cell properties in breast cancer by down-regulating PTEN^26^. In accordance with these previous studies, we also show that in ECFC derived from peripheral blood, PTEN was down-regulated by mature miR-21 and that this is correlated with the induction of a stem cell phenotype.

Numerous pathological conditions have been reported to lead to dysfunction of endothelial progenitors due to a senescent phenotype. Thus, endothelial progenitor cells from smokers and patients with chronic obstructive pulmonary disease patients or from patients with severe type 2 diabetes are mostly senescent and display reduced expression of Sirt-1^27,28^. Patients with PAD also represent a population of patients with a high level of senescent progenitor cells. In this regard, the fact that sCD146 reduces the number of senescent cells through a reduction in p21 and p16 expression and an increase in Sirt1 is of interest. This effect only occurs in sub-confluent cells with a limited number of passages, showing that culture conditions are important for obtaining functional ECFC. When cultured in these conditions, in the presence of sCD146, pb-ECFC cells are not more senescent than cb-ECFC, which are considered as a good therapy product. Likewise, pb-ECFC-sCD146 maintain a higher proliferative capacity than pb-ECFC after several passages, although it appears, as observed for senescence, that it is better to use them at early passages.

Considered altogether, our results suggest that the treatment of pb-ECFC with sCD146 during the amplification step in culture allows maintaining cells in an immature state favouring a robust proliferative potential and a low senescent phenotype compatible with obtaining large amounts of cells to be grafted. Once injected into the animals and grafted into the ischaemic area, we may assume that cells may differentiate into more mature endothelial cells able to participate in the vascular regeneration by incorporating, even at a low level, to the vessels and/or secreting angiogenic factors. Although various cell types have been shown to promote revascularization, and in particular iPS, it appears that ECFC constitute a bona fide endothelial progenitor with a demonstrated therapeutic efficacy in different preclinical models of ischaemia, including myocardium^29^, brain^30^, hind limb^31^, kidney^32^ and retinal disease where important effects where observed^33–36^, paving the way for using these cells in patients.

Of interest, the effects of sCD146 on peripheral blood ECFC stem cell properties and senescence occur through the induction of miRNA. miRNAs have emerged as key players in many biological processes, including cell proliferation, differentiation and apoptosis^37^. In our study, we show that mature miR-21 plays a key role in stem cell properties maintenance, the endothelio-mesenchymal transition and senescence prevention. Thus, sCD146 enhances mature miR-21 that is involved in oct4 induction and in the generation of a partial mesenchymal phenotype. In addition, mature miR-21 is involved in the prevention against ECFC senescence. Mature miR-21 thus appears to play a central role to coordinate the different actions of sCD146. In a recent study, Richart *et al*. have shown that mature miR-21 coordinates human multipotent cardiovascular progenitor therapeutic potential. They have shown in particular that mature miR-21-deficient mice displayed a defective angiogenic phenotype and that this miRNA was important for endogenous post-ischaemic revascularisation through its effects on progenitor cells^12^. As already mentioned by these authors, the role of mature miR-21 in angiogenesis and cell survival is controversial. Indeed, some studies report that mature miR-21 induces angiogenesis through Akt, Erk and HIF-1 alpha^38^ whereas other studies report that mature miR-21 expression is correlated with dysfunction of angiogenic progenitor cells^39,40^. In their study, Richart *et al*. concluded that the biological activity of mature miR-21 relied on the tuning of the balance between the type of cells and the homeostasis of the ischaemic tissues. Likewise, the effect of mature miR-21 on cell survival is also controversial. Thus, Zhu *et al*. showed that mature miR-21 enhanced endothelial cell senescence^41^, whereas Tu *et al*.^42^ showed that mature miR-21 played a protective role in myocardial cell survival through the Pten/Akt signalling pathway. In ECFC isolated from peripheral blood, our study clearly shows that sCD146 acts, at least in part, through mature miR-21 and that this miRNA displays positive effects on the regenerative properties of these cells.

The soluble CD146 peptide thus appears as an interesting factor able to promote the sorting of ECFC from peripheral blood and to maintain both their stem cell phenotype and their survival capacity. Once injected in ischaemic tissues, these cells display interesting regenerative properties for cell therapy procedures. As a proof of concept, we used sCD146 for the sorting and amplification of ECFC from patients with PAD. The results thus show that it is possible to isolate, amplify and re-inject these cells by starting from a low volume of peripheral blood, typically 40 ml. These cells display therapeutic properties since they are able to induce re-vascularization in an animal model of hindlimb ischaemia in mice.

In conclusion, our study shows that ECFC can be easily isolated and amplified from peripheral blood of patients with peripheral arterial disease by using sCD146 in the isolation and culture media. The presence of the molecule allows amplifying the number of colonies and their growth. In addition, it allows maintaining the cells with a stem cell phenotype, as demonstrated by the expression of embryonic transcription factors, and maintaining a non-senescent phenotype. The use of sCD146 appears thus to be of major interest, not only with ECFC but also probably with other cellular fractions, such as bone marrow-derived CD34 + cells, mesenchymal stem cells or the stromal vascular fraction, with the objective of developing therapies based on autologous stem cells for the treatment of ischaemic cardiovascular diseases.

## Methods

### Patients

Four patients with peripheral arterial disease (PAD) were included in this study. The clinical characteristics of patients are detailed in Supplementary Table 1. ECFC were isolated from a sample of whole blood taken from eight heparinized tubes (40 ml) and treated with 50 ng/ml of recombinant sCD146 (Biocytex, Marseille, France) throughout the culture period.

### Isolation and culture of ECFC from adult peripheral and umbilical cord blood samples

Whole blood samples (250–350 ml) were collected in citrate phosphate dextrose (CPD) solution from 23 healthy human donors (13 men, 10 women; age range: 18–34 years). Buffy coats (35–40 ml) were collected in CPD solution from 16 healthy human donors (7 men, 8 women; age range: 18–31 years). Whole blood and buffy coat were provided by the Etablissement Français du Sang (EFS, Marseille, France), in compliance with French legislation.

Human umbilical cord blood samples (20–50 ml) from 6 healthy newborns (≥37 weeks) were collected in CPD solution. These samples were provided by the Assistance Publique Hôpitaux de Marseille, and all parents gave written informed consent for the use of cord blood in accordance with the Declaration of Helsinki.

ECFC were isolated from the mononuclear cell fraction (MNC) obtained from the whole blood, buffy coat and umbilical cord blood. To this end, MNCs were plated at 5 × 10^6^ cells/well in 6-well plates and cultured as described by Ingram *et al*.^43^.

For the MNCs obtained from whole blood or buffy coat, the cells were divided into two equal quantities and one part was treated with 50 ng/mL of recombinant sCD146 (Biocytex, Marseille, France) resuspended in PBS (Phosphate Buffered Saline) in order to compare the number of colonies, their time of onset and their area in culture.

Colonies of endothelial cells appeared between 14 and 28 days of culture and were identified as well-circumscribed monolayers of cobblestone-appearing cells. ECFC were enumerated by visual inspection using an inverted microscope (Nikon) under 20X/0.45 magnification. A monolayer containing at least 20 cells with a cobblestone pattern was counted as a colony.

### Flow cytometry analysis

Early passage (2–4) ECFC derived from peripheral blood were resuspended in 100 μl of 1X PBS containing 0.1% bovine serum albumin (BSA) at a concentration of 300,000 cells/mL and analysed by flow cytometry. The presence of several markers was analysed by incubation with specific antibodies. We used mouse monoclonal primary antibodies directed against human CD34 coupled with Fluorescein isothiocyanate (FITC), human CD146 coupled to phycoerythrin (PE), human VEGFR2 coupled to tandem PE-cyanine7 (PC7), human CD133 coupled to allophycocyanin (APC), and human CD45 coupled to the allophycocyanin-alexa750 (APC A750) tandem. For isotypic controls, we used mouse IgG1 coupled to FITC, APC A750 and PC7, APC coupled IgG2b and PE-coupled IgG2a. Each preparation was incubated for 30 minutes at 4 °C. After three washes in PBS 1X and 0.1% BSA, the fluorescence intensity was analysed by flow cytometry (Gallios TM Flow Cytometer, Beckman Coulter, Villepinte).

### Capillary tube formation

ECFC incubated or not with 50 ng/ml of recombinant human sCD146 were cultured at 20 000 cells per well in 96-well plates coated with 50 μl of growth factor-reduced Matrigel (10 mg/ml; BD Biosciences). Capillary-like structures were counted using ImageJ software (NCBI). Each experiment was done in triplicate.

### Spheroid-based sprouting assay

ECFC were seeded as previously described by Korff *et al*.^44^ and incubated or not with 50 ng/mL of recombinant sCD146. After 30 minutes, 100 μl of EBM2 containing or not 50 ng/mL of recombinant sCD146 was added to each well. After 24 hours, 20 spheroids were analysed for each independent sample using a Leica microscope at a 20x magnification (Leica sp5, Leica, Nanterre, France). Cumulative sprout length, number of sprouts, and ramifications were quantified using ImageJ software.

### Cell proliferation assay

ECFC were seeded on 96-well plates (5.10^3^/well) and cultured in EGM2-MV medium for 24 hours. After treatment, cell proliferation was assayed using the BrdU Labeling and Detection Kit III (Roche Corporation) as indicated by the manufacturer. The results were expressed as arbitrary units. Experiments were performed in triplicate.

### SA-β-gal staining

Senescence-associated-β-galactosidase (SA-β-gal) activity was analysed using a senescence detection kit (BioVision Research Products) according to the manufacturer’s instructions. The percentage of SA-β-gal–positive cells was counted in 10 randomly selected microscopic fields (magnification 20x; 400–600 cells).

### RNA isolation and quantitative RT-PCR

For analysis of mature miR-21 expression, total RNA was extracted using the miRNeasy Mini Kit (Qiagen) following manufacturer’s instructions.

For all other analyses, total RNA was extracted using the RNeasy Mini Kit (Qiagen) following the manufacturer’s instructions. RNA concentration was obtained with a Nanodrop apparatus (Thermo Scientific). Reverse transcription was realized using the High-Capacity cDNA Reverse Transcription (Life Technologies) or Taqman MicroRNA Reverse Transcription Kit (Applied Biosystems). The quantitative polymerase chain reaction was performed using Sybergreen (Life Technologies) or Taqman Universal Master Mix II, no UNG (Applied Biosystems, Stratagene) and primers corresponding to the cDNA studied.

The sequences or references of primers are given in Table 1.

**Table 1:** Sequences or references of the primers.

Primers	Right	Left
GADPH	5′-GGTGGTCTCCTGACTTCAACA	5′-GTTGCTGTAGCCAATTGTTGT
Amot	5′-TCTGCTCCTGCTCAGACTCA	5′-ACAGGCCCATCTGTTTTGTC
VEGFR2	5′-TGTGGGTTTGCCTAGTGTTTCT	5′-CACTCAGTCACCTGCACCCTT
eNOS	5′-CTCATGGGCACGGTGATG	5′-ACCACGTCATACTCATCCATACAC
VE-CADH	Hs00170986_ml (AppliedBiosystem)	
PECAM-1	Hs00169777_ml (AppliedBiosystem)	
α-SMA	Hs00426835_gl (AppliedBiosystem)	
VIMENTIN	Hs00185584_ml (AppliedBiosystem)	
PTEN	Hs02621230_s1 (AppliedBiosystem)	
OCT-4	Hs04260367_gh (AppliedBiosystem)	
SOX-2	Hs01053049_s1 (AppliedBiosystem)	
NANOG	Hs02387400_g1 (AppliedBiosystem)	
KLF-4	Hs00358836_m1 (AppliedBiosystem)	
c-MYC	Hs00905030_m1 (AppliedBiosystem)	
hsa-miR-21	000397 (AppliedBiosystem)	
U6	001973 (AppliedBiosystem)	
RPL13	Hs00744303_s1 (AppliedBiosystem)	

### Western-blot

Western-blot analysis was performed as previously described^45^. Briefly, ECFC were grown on plates treated or not with 50 ng/mL sCD146, then washed in PBS, scraped off the plates and lysed with 200 μl denaturing lysis buffer (150 mM NaCl, 10 mM TrisHCl pH 8, 1 mM EDTA, NP40 10% and protease inhibitors) for 10 minutes at 4 °C. After centrifugation (12,000 g, 10 minutes, 4 °C) to eliminate cell debris and nuclei, proteins were quantified by protein assay (BCA Protein Assay Kit, Pierce). Then, 30 μg of protein were mixed with NuPAGE lithium dodecylsulfate (LDS) sample buffer (Invitrogen) and NuPAGE sample-reducing agent (Invitrogen). The samples were separated by electrophoresis on 4–12% gradient NuPage SDS-polyacrylamide gels(Life Technologies). After transfer, membranes were blocked with BSA 4% and incubated with a primary antibody and a secondary antibody coupled to peroxidase before detection with the ECL kit (Pierce).

Antibodies against p16^INK4a^, p21^WAF^, SIRT1, p53, VE-Cadherin, α-SMA, Vimentin and Actin were purchased from Cell Signaling Technology. Antibodies against PECAM-1, Oct-4, Sox-2, KLF-4 and c-MYC were purchased from Santa Cruz Biotechnology. The antibody against Nanog was purchased from R&D systems. All antibodies were used at the recommended dilution for immunoblotting.

### Stem Cell-Associated miRNA Plate Array

The Stem Cell-Associated miRNA Plate Array (Signosis) was used as described by the manufacturer. Briefly, total RNA from peripheral blood ECFC treated or not with 50 ng/mL sCD146 was extracted using the miRNeasy Mini Kit (Qiagen) following the manufacturer’s instructions. Then, a mixture containing 30 μg of RNA, hybridization buffer and Biotin Detection Oligo was prepared, and 100 μl of hot aliquot was deposited in each well of the miRNA plate array. The quantification was carried out with a luminometer (Tecan Infinity F200).

### miRNA and transfection experiments

For miRNA experiments, peripheral ECFC treated or not with 50 ng/mL sCD146 were transfected with control or anti-miR-21 (inhibitory molecules of mature miR-21. Applied Biosystems) at a 100 nM concentration using magnetofection Silence Mag (OZ Biosciences) as indicated by the manufacturer’s instructions. Approximately 85–90% ECFC transfection efficiency was achieved. Transfected cells were used 48 hours after transfection for *in vitro* studies.

### Induction of hindlimb ischaemia in nude mice

Eight-weeks-old male Nude mice were housed in enriched cages placed in a temperature-and hygrometry-controlled room with daily monitoring and fed with water and commercial diet ad libitum. Unilateral hindlimb ischaemia was performed after femoral artery excision under 2% isoflurane anaesthesia. In a first series of experiments, after surgery, animals were split in different treatment groups: one control group injected retro-orbitally with PBS, one group injected retro-orbitally with peripheral blood ECFC (pb-ECFC), one group injected with peripheral blood ECFC from the same clone than pb-ECFC and treated throughout the amplification procedure with 50 ng/ml sCD146 (pb-ECFC-sCD146) and one group injected with ECFC derived from cord blood (cb-ECFC). In experiments performed with patients with PAD, two groups were used: one control group injected retro-orbitally with PBS and one group injected with ECFC from peripheral blood of PAD patients sorted in the presence of sCD146.

### Laser Doppler blood flow analysis

Laser Doppler perfusion imaging (Perimed, Craponne, France) was used to assess re-vascularisation from day 1 to day 15 after surgery. Perfusion results are expressed as a ratio of ischaemic to non-ischaemic limb blood flow.

### Angiography CT imaging

The animals were deeply anaesthetised with a mix of Ketamine/Xylasine (100 mg/kg and 10 mg/kg respectively) and intracardiac infused with 2 ml of lipiodole. CT 3D images were obtained with a microPET/microCT rodent model scanner (nanoPET/CT®, Mediso) with characteristics below 70 kVp energy, exposure time of 300 ms and 720 projections.

### Statistical analysis

Data were expressed as the mean ± SEM of the indicated number of experiments. Statistical analysis was performed with Prism software (GraphPad Software Inc., San Diego, CA). Significant differences were determined using the non-parametric Mann-Whithney U test.

### Ethical approval and informed consent

Research on humans was approved by the local ethic committee Assistance Publique-Hôpitaux de Marseille, and all patients gave their written informed consent. Methods involving human participants were performed in accordance with the relevant guidelines and regulations.

All procedures using animals were approved by the Institution’s Animal Care and Use Committee (CE14, Aix-Marseille University) and were conducted according to the EU Directive 2010/63/EU and the recommendations of the Helsinki Declaration.

### Data availability

All data generated or analysed during this study are included in this article (and its Supplementary Information files).

## Electronic supplementary material

Supplementary information
