# Supplementary material for: Stem cell properties of peripheral blood endothelial progenitors are stimulated by soluble CD146 via miR-21: potential use in autologous cell therapy
Source: Sci Rep. 2018 Jun 20;8:9387. doi: 10.1038/s41598-018-27715-4 (PMC6010456; doi:10.1038/s41598-018-27715-4)
Supplement: Supplementary file 1 — Supplementary information [file 41598_2018_27715_MOESM1_ESM.pdf]

SUPPLEMENTARY INFORMATION

Stem cell properties of peripheral blood endothelial progenitors are stimulated by soluble CD146 via miR-21: potential use in autologous cell therapy

Amel Essaadi, Marie Nollet, Anaïs Moyon, Jimmy Stalin, Stéphanie Simoncini, Laure Balasse, Alexandrine Bertaud, Richard Bachelier, Aurélie S. Leroyer, Gabrielle Sarlon, Benjamin Guillet, Françoise Dignat-George, Nathalie Bardin, and Marcel Blot-Chabaud

Characteristics of patients with PAD	Patient N°1	Patient N°2	Patient N°3	Patient N°4
Age (years)	83	49	65	91
Sex	F	M	M	M
Volume of blood samples (ml)	40	40	40	40
Number of MNC	26.10^{6}	27.10^{6}	30.10^{6}	22.10^{6}
Number of ECFC colonies	1	1	1	No
Number of injected cells	100000	100000	100000	No cell
Onset time (days)	16	17	23	-
Time to injection (days)	30	30	30	-

Supplementary table 1: Characteristics of patients with Peripheral Arterial Disease (PAD)

The characteristics of patients and of ECFC isolated from 40 ml of blood are given.

A

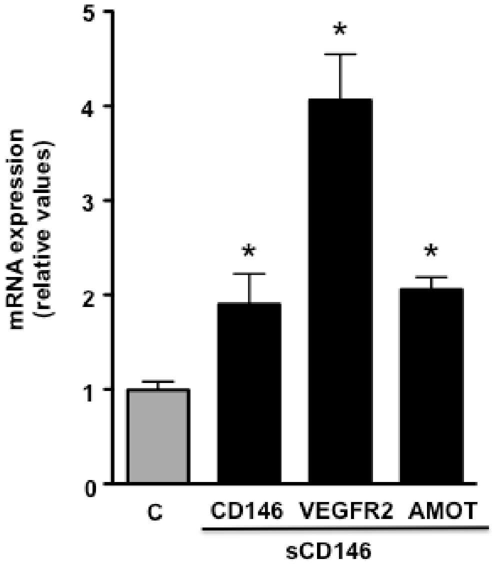

B

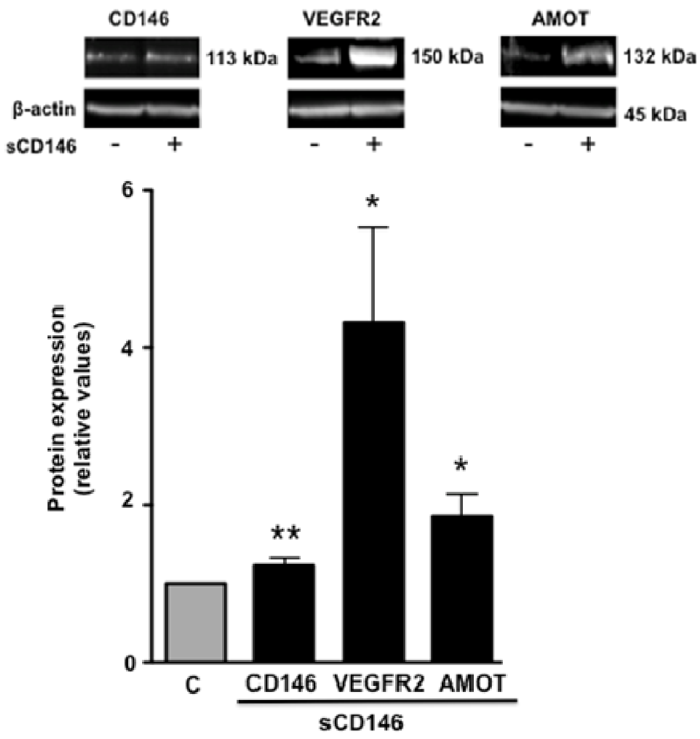

Supplementary Figure 1: Soluble CD146 enhances the expression of membrane CD146, angiomotin and VEGFR2 in pb-ECFC

A: Effect of 48 hours of treatment with 50 ng/ml sCD146 on the mRNA expression of CD146, angiomotin p80 and VEGFR2.

B: Effect of 48 hours of treatment with 50 ng/ml sCD146 on the protein expression of CD146, angiomotin p80 and VEGFR2. The same β-actin was used for CD146 and Amot because the membrane was stripped and both CD146 and Amot were detected on the same blot.

*, **: P<0.05, 0.01, experimental *vs* control

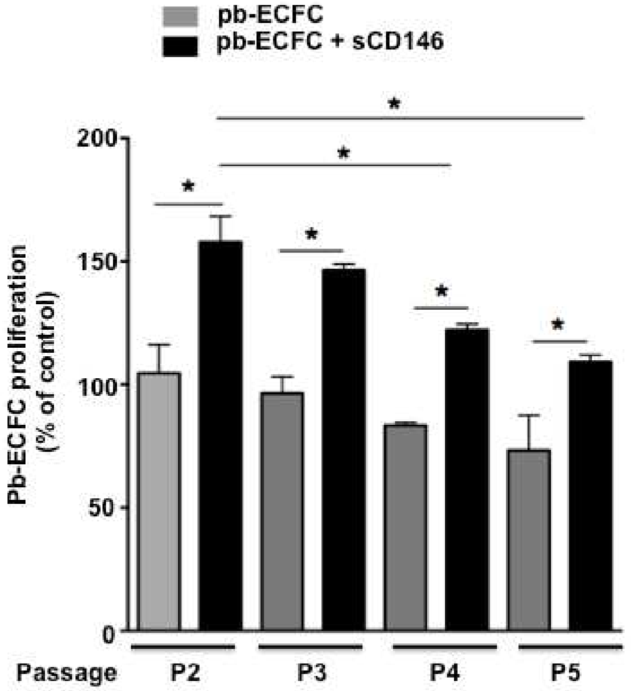

Supplementary Figure 2: Soluble CD146 maintains the proliferative capacity of pb-ECFC in culture

Comparison between the proliferative capacity of pb-ECFC and pb-ECFC treated with sCD146 50 ng/ml at different passages in culture.

*****: P<0.05, experimental *vs* control

A

Up-regulated miRNA

1	Let7b	11	mir 26 a	21	mir 125b
2	Let7c	12	mir 27a	22	mir 126
3	mir 1	13	mir 30a-3p	23	mir 128a
4	mir 9	14	mir 34a	24	mir 132
5	mir 10a	15	mir 99a	25	mir 133a
6	mir 15b	16	mir 101a	26	mir 141
7	mir 17-5p	17	mir 103	27	mir 149
8	mir 19 a	18	mir 106a	28	mir 150
9	mir 19 b	19	mir 107	29	mir 181a
10	mir 21	20	mir 125a	30	mir 218

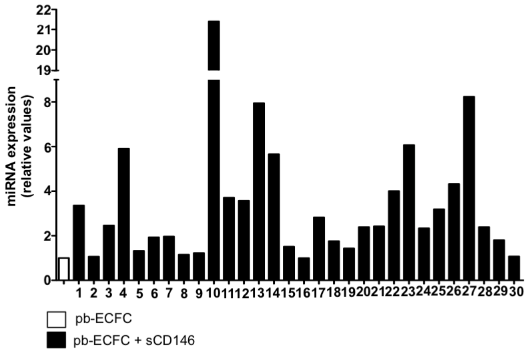

B

Down-regulated miRNA

1	mir 15a
2	mir 23a
3	mir 24
4	mir 93
5	mir 96
6	mir 124a
7	mir 135b
8	mir 146a
9	mir 155

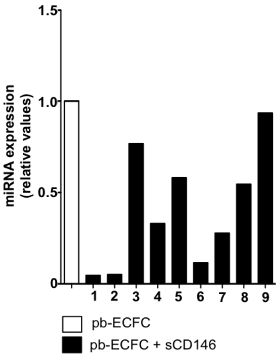

Supplementary Figure 3: Effect of soluble CD146 on miRNA expression

A Stem Cell-Associated miRNA Plate Array was used to evaluate sCD146 effects.

A: Effect of 48 hours of treatment with 50 ng/ml soluble CD146 on miRNA expression in pb-ECFC.

The miRNA that were up-regulated are indicated, and the effect was quantified.

B: Effect of 48 hours of treatment with 50 ng/ml soluble CD146 on miRNA expression in pb-ECFC.

The miRNA that were down-regulated are indicated, and the effect was quantified.

Figure 3

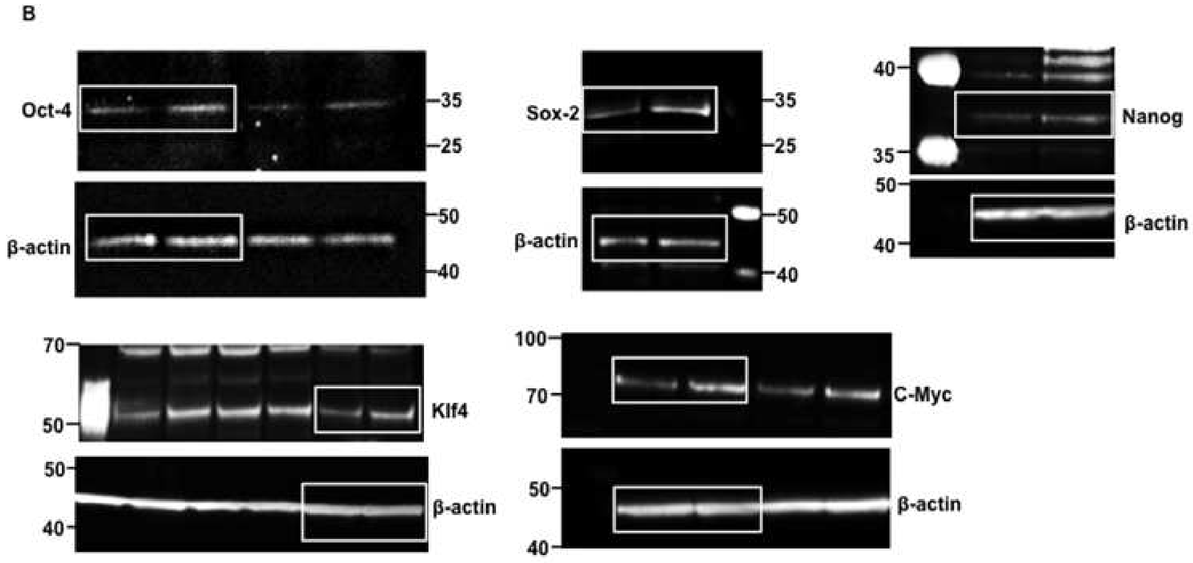

Figure 4

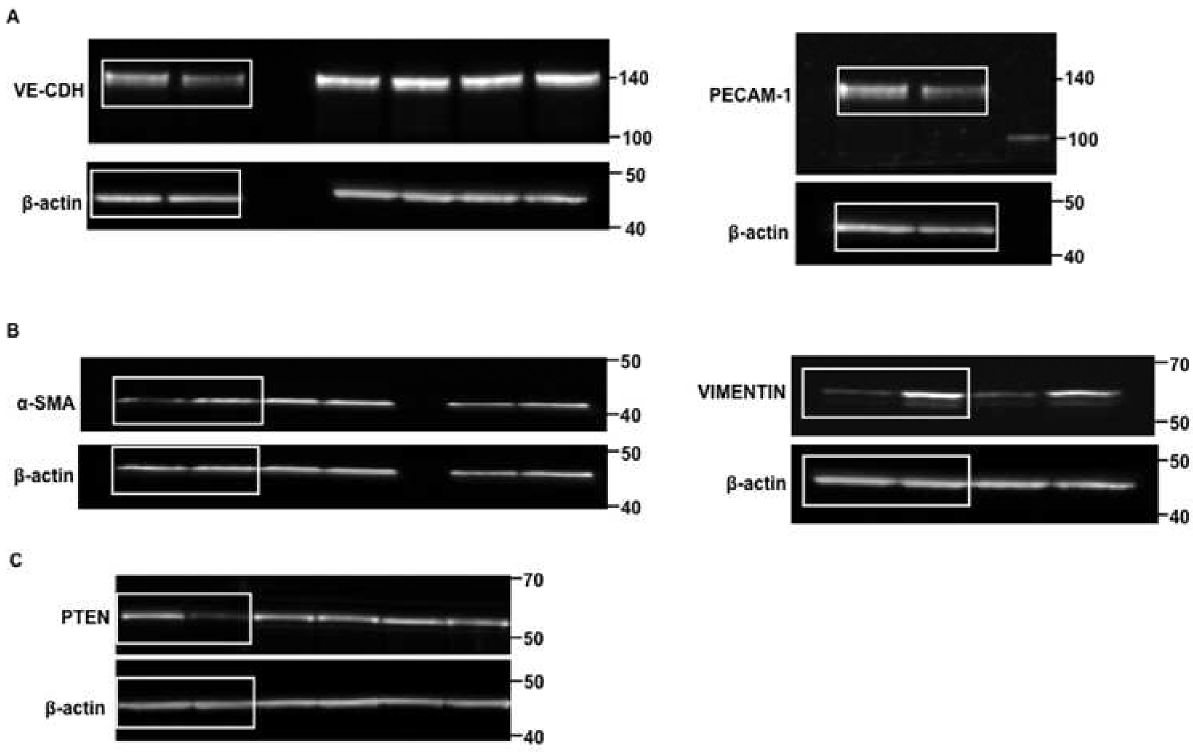

Supplementary Figure 4: Raw data of western-blots

Figure 5

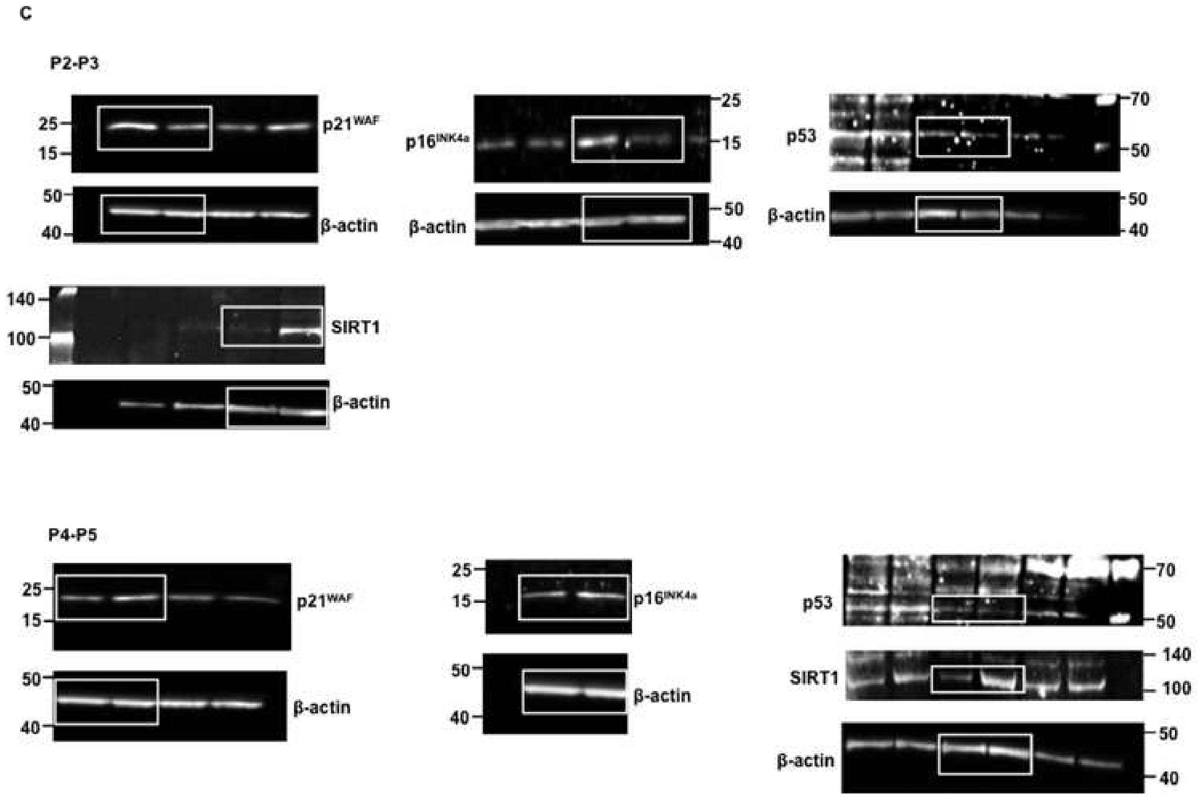

Figure 6

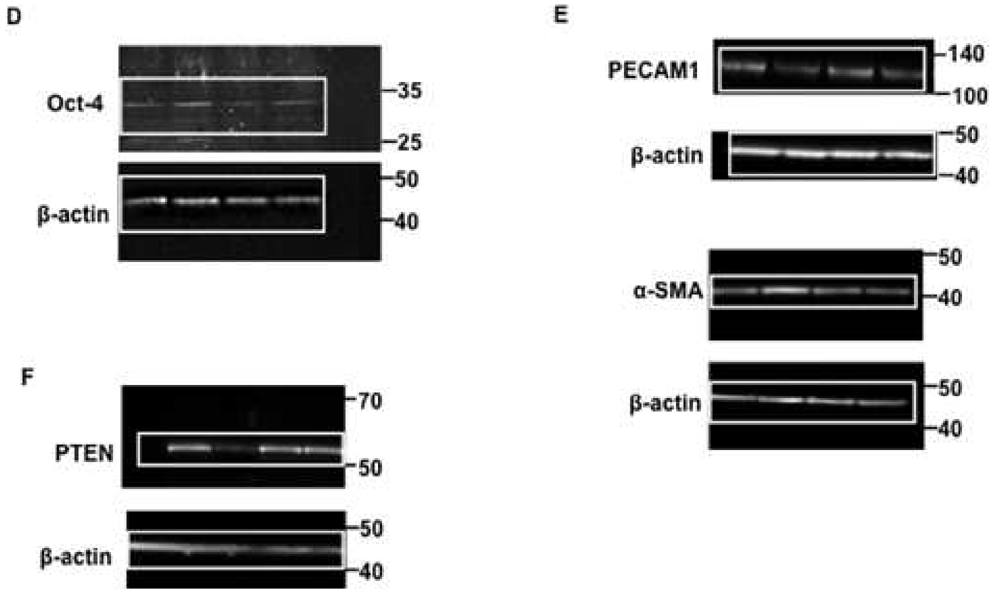

Supplementary Figure 4 (continued)

Supplementary Figure 1

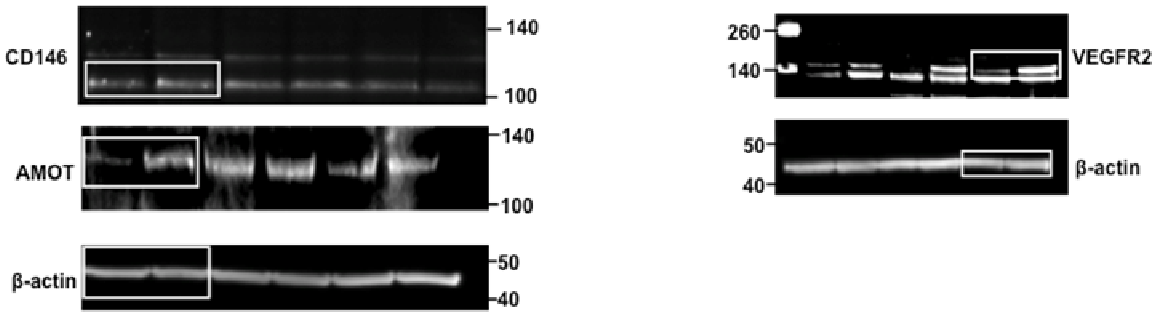

Supplementary Figure 4 (continued)
